# Possible Roles of Vitamin D in Bone Grafting

**DOI:** 10.7759/cureus.14688

**Published:** 2021-04-26

**Authors:** Georgios Markopoulos, Panagiotis Lepetsos, Despina N Perrea, Dimitrios C Iliopoulos, Vasileios S Nikolaou

**Affiliations:** 1 Second Department of Orthopaedics, National and Kapodistrian University of Athens School of Medicine, Athens, GRC; 2 Department of Trauma and Orthopaedics, Athens Medical Center, Athens, GRC; 3 Laboratory of Experimental Surgery and Surgical Research, National and Kapodistrian University of Athens School of Medicine, Athens, GRC

**Keywords:** vitamin-d deficiency, vitamin-d, vitamin d supplementation, bone graft substitutes, osseointegration

## Abstract

Bone grafting is one of the most commonly used options to treat large bone defects. Evidence has shown that vitamin D may affect osseointegration, a major component for successful bone grafting. In vitro studies have proved that implants coated with activated vitamin D stimulate bone production and reduce bone resorption around implants. Animal studies have noticed that oral administration of vitamin D may stimulate bone formation as well as strengthen and support the interaction between bone and implants. Vitamin D insufficiency may affect negatively the cortical peri-implant bone formation, suggesting a negative effect in graft incorporation. Few clinical studies have observed that vitamin D administration enhanced graft incorporation and bone formation, while severe vitamin D deficiency is associated with failed implant osseointegration. Even though there are encouraging results of vitamin D supplementation on graft incorporation in animal studies, the use of vitamin D as an adjuvant in bone grafting procedures cannot be fully supported at the moment. However, there is theoretical support in the use of vitamin D after surgery and the use of bone grafts to support the bone structure, relieve pain and increase graft absorption. Further experimental and clinical studies are required to support the administration of vitamin D and its analogues in such cases.

## Introduction and background

Vitamin D is a fat-soluble vitamin regulating calcium and phosphorus metabolism in tissues. The term vitamin D includes both vitamin D2 (ergocalciferol) and, most importantly, vitamin D3 (cholecalciferol). Most of the needs of the human body for vitamin D (about 90%) are covered by the endogenous composition of the vitamin under the influence of ultraviolet radiation, while the rest is covered by nutritional intake. When sunlight hits healthy skin, it converts 7-dihydrocholesterol to provitamin D3 and then, through a rapid reaction to its isomer, vitamin D3 or cholecalciferol. Both D2 (ergocalciferol) and D3 (cholecalciferol) are hydroxylated in the liver at position 25 and converted to 25-hydroxyvitamin D [25-(OH)-D]. This is followed by a new hydroxylation, in position 1, which occurs mainly in the kidneys, under the effect of the enzyme 1α-hydroxylase, resulting in the formation of the active metabolite of vitamin D, called calcitriol (1, 25-dihydrocholecalciferol or 1,25-(OH)2-D). The level of vitamin D production is regulated in response to the serum levels of calcium, phosphorus and parathyroid hormone (PTH) [1,2].

Effects of vitamin D

Cellular actions of vitamin D are mediated by binding to the vitamin-D receptor (VDR), a class II intracellular steroid expressed in all nucleated cells. In the gastrointestinal tract, active vitamin D promotes the absorption of calcium and phosphorus. In bone, at increased concentrations, it stimulates osteoclastic activity, leading to an increase of calcium release into the circulation. Moreover, it augments the osteoblastic production of extracellular matrix proteins. In the kidneys, along with the PTH, active vitamin D can further stimulate the reabsorption of calcium. In the parathyroid glands, vitamin D inhibits the release and secretion of PTH [3,4].

Vitamin D deficiency affects a very large percentage of the world population and is determined by serum 25-(OH)-D levels. Vitamin D deficiency may result from inadequate dietary intake and metabolic disorders along with inadequate exposure to sunlight. This deficiency is associated with catabolic bone turnover, bone loss and osteoporosis. In vivo studies have shown that the administration of vitamin D and its precursor, alfacalcidol, can be used in the treatment of osteoporosis, as vitamin D suppresses bone resorption by osteoclasts. Daily in vivo administration of vitamin D inhibits osteoclast differentiation by suppressing RANKL (receptor activator of nuclear factor-kappa-Β ligand) expression in osteoblasts [5,6].

## Review

Role of vitamin D in bone healing

The role of vitamin D in bone healing has been under investigation for several years. Vitamin D deficiency has also been associated with impaired and delayed callus formation and fractures healing; however, the role of vitamin D has not been clarified [7].

Several animal studies have been published in an attempt to elucidate the role of vitamin D in bone healing. The first study was published in 1981 and showed that calcitriol administration improved healing biomechanical properties in male rats with drill-induced femoral defect [8]. In 1997, Omeroğlu et al. observed that an administration of a single high-dose of vitamin D led to improved callus formation and mineralization in male guinea pigs with a tibial fracture [9]. In a more recent animal study, performed in ovariectomized rat models with fractured femurs, authors found that daily oral administration of calcitriol resulted in significantly increased bone biomechanical strength and better callus remodelling [10]. In another study, in a rat model, authors concluded that low vitamin D may negatively affect early healing without clarifying the exact mechanism of this action [11]. On the contrary, Fügl et al., in an animal study, concluded that vitamin D insufficiency does not impair bone regeneration in the rat jaw and local vitamin D application does not promote healing [12].

Human studies have shown that vitamin D deficiency at the time of trauma is associated with a higher incidence of delayed union [13]. Moreover, although the function of VDRs has been thoroughly studied, the role in the inter-organ communication between skeletal, nervous and bone marrow, need further attention [14]. By affecting both osteoclasts and osteoblasts, vitamin D regulates the process of bone formation. Given that bone formation is of paramount importance for graft incorporation, one could infer its significance in the use of bone graft through surgical procedures [5,6,15,16].

Role of vitamin D in bone grafting

The role of vitamin D in bone regeneration is well established. Bone grafting is one of the most commonly used options to treat large bone defects. A bone graft is defined as a material with specific properties, including osteoinduction, osteoconduction, and osseointegration. Osteoinduction is the process of recruitment and stimulation of immature cells to differentiate into pre-osteoblasts. Osteoconduction is the ability of osteoblasts to move across a scaffold in the grafting area and slowly replace it with new bone over time. Osseointegration is defined as the ability of an implant to anchor with bone formation at the bone-implant interface without the formation of soft tissue [17].

During the osseointegration period, vitamin D can affect the result through its effect on the immune system. According to some epidemiological studies, calcitriol can affect both innate and acquired immunity by promotion of macrophage function, enhancement of chemotaxis and phagocytosis, and production of cytokines and other immunomodulatory peptides [18]. The association of vitamin D deficiency with reduced antimicrobial activity of cells and increased risk of bacterial infections is reported by many studies and indicates the importance of vitamin D adequacy at the clinical level both during osteointegration and at the later stages of graft union [19].

Unfortunately, literature is scarce regarding evidence for the role of vitamin D in bone grafting procedures. The process of implant osseointegration is similar to bone grafts incorporation and depends on patient-related factors, including bone quality and quantity and the host response. As bone regeneration affects the osseointegration of dental implants, there may be a possible correlation between vitamin D levels and the bone grafting process through osseointegration in dental implants [20]. However, there are only preclinical studies about vitamin D deficiency and bone regeneration, showing that administration of vitamin D may have a beneficial effect on bone turnover. The majority of in vivo studies report a positive correlation between vitamin D and bone mineral density of the newly formed bone around the implant through the induction of osteoblastic activity, inhibition of osteoclasts and reduced risk of bacterial infection [21-24]. However, it should be taken into account that most of them involve experimental models, mainly rats and not humans.

In vitro studies

In vitro studies by Satué et al. have shown that ultraviolet radiation of titanium implants coated with 7-dihydrocholesterol stimulates osteoblastic differentiation and vitamin D production [25], inhibit osteoclastogenesis [26], promote gene expression of bone formation markers and alkaline phosphatase activity [27], reduce extracellular matrix breakdown [28] and enhance the osteogenic differentiation of mesenchymal stem cells [29], around the implant.

Animal studies

Animal studies have noticed that administration of vitamin D may stimulate bone formation as well as strengthen and support the interaction between bone and implants. Beyond the regulation of bone formation, vitamin D affects immune response in the field of osteoimmunology and may potentially influence early implant healing. Through the alteration of osteoclast function, low levels of vitamin D may affect the balance between the immune system and bone metabolism during implant healing [19].

In 2002, Merida et al. conducted an experimental study investigating the effects of a vitamin D analog on bone reconstruction by vascularized bone allograft. The authors concluded that the addition of 22-oxa-1,25-dihydroxyvitamin-D3 enhanced bone graft union by exerting an anabolic action on bone reconstruction [30]. Another animal study by Kaya et al., in 28 male rats, showed that oral calcium and vitamin D supplementation accelerated bone regeneration in alloplastic bone-grafted tibial defects [31]. In addition, incorporating biodegradable polyurethane bone graft substitutes with vitamin D3 could enhance bone regeneration of bicortical defects in the iliac crest of oestrogen-deficient sheep. The structure of the newly formed cancellous bone was radiographically and histologically similar to the native bone [32].

A study by Kelly et al. showed that vitamin D insufficiency in rats may compromise the osseointegration of Ti6Al4V implants [22]. Mengatto et al. demonstrated impaired osseointegration in rats with vitamin D deficiency [33]. After the implantation of titanium screws in the osteoporotic rat tibia, Zhou et al. examined the effect of vitamin D administration. They found increased bone assimilation and improved osseointegration in osteoporotic rats after administration of calcitriol [34]. Working on ovariectomized rats, Dvorak et al. observed that vitamin D deficiency may affect negatively the cortical peri-implant bone formation causing a decrease in bone-to-implant contact, which can be compensated by a vitamin D [21]. Similar were the results of another study by Wu et al., who noticed increased bone-to-implant contact in diabetic rats after the combined administration of insulin and vitamin D [24]. In mice with chronic kidney disease, vitamin D injections enhanced the fixation of titanium implant insertion into the distal end of the femur [23].

Another study by Salomo-Coll et al. investigated the osseointegration of titanium implants supplemented with vitamin D in post-extraction sockets of six dogs. Authors found that locally applied vitamin D may increase the bone-to-implant contact by 10% with a three-month follow-up [35]. Cho et al. investigated the reaction of bone tissue to anodized titanium implant surfaces coated with a poly(D,L-lactide-co-glycolide) (PLGA) solution mixed with calcitriol in rabbits tibias. Authors reported the formation of micro-particles, next to the implant surface, which may stimulate bone formation [36]. Another preclinical study in dogs showed that local administration of vitamin D stimulated new bone formation, increased bone density and improved implant stability around surgically created sockets in the mandible [37]. In another experimental study in rabbits, 28 implants inserted in the tibia and coated with 1,25-(OH)2D3 demonstrated a trend for better osseointegration in comparison with uncoated implants [38].

Human studies

Human studies are required in order to prove that sufficiency or supplementation of vitamin D may assist the incorporation of bone grafts postoperatively [39]. In 2016, Schulze-Späte et al. published the results of a randomized, double-blind, placebo-controlled clinical study on systemic vitamin D administration and local bone formation in maxillary sinus augmentation. The intervention group received 5000IU vitamin D3 with 600 mg calcium while the placebo group received only calcium. Although increased vitamin D levels were observed in the intervention group, with possible benefits in terms of bone formation, authors found no statistically significant importance in bone formation or graft resorption among the studied groups [40]. However, the effect of vitamin D in the graft incorporation cannot be excluded so easily. Since the absorption of bone grafts and its replacement with bone tissue require the activation of osteoclasts, a theoretical model would infer that vitamin D would inhibit the initial phase of bone graft absorption [41].

A comparative study by Amr et al. evaluated the effect of vitamin D3 when mixed with xenogenic bone grafts in alveolar ridge augmentation performed simultaneously with implant placement for the treatment of bone peri-implant defects. The authors concluded that vitamin D improved implant stability and enhanced graft incorporation and bone formation [42].

A large retrospective study published by Guido Mangano et al. investigated the association of low serum levels of vitamin D with early dental implant failure. The study population was divided into three groups according to serum levels of vitamin D. Similarly with the aforementioned study, no significant difference in implant failure was found between the three groups, even though the highest incidence was observed in severe vitamin D deficiency (<10 ng/ml). Authors suggested that administration of vitamin D in the weeks before a dental implant may be useful, especially in patients with severe vitamin D deficiency and vitamin D supplementation should be maintained until the required serum levels are achieved [43]. Recently, the same research team published the results of another retrospective study, confirming the conclusions from their previous study. Patients with normal serum levels of vitamin D reported low failure rates, while severe vitamin D deficiency was associated with a fourfold increase of implant failure. However, no statistical significance was observed (p = 0.105) [44].

In a case-control study, no association was found between single nucleotide polymorphisms of the VDR gene and implant failure in a Brazilian population [45]. Fretwurst et al. reported two cases of early dental implant failure where crestal bone grafting with autologous material was used. Both patients were found to suffer from severe vitamin D deficiency. After the restoration of vitamin D levels, dental implantation was successful in both patients [46]. Bryce and MacBeth reported a case of a failed osseointegration of a dental implant in a 29-year old with a severe deficiency of vitamin D [47].

## Conclusions

Even though there are encouraging results of vitamin D supplementation on graft incorporation in animal studies, the use of vitamin D as an adjuvant in bone grafting procedures cannot be fully supported at the moment. However, there is theoretical support in the use of vitamin D after surgery and the use of bone grafts to support the bone structure, relieve pain and increase graft absorption. Further experimental and clinical studies are required to support the administration of vitamin D and its analogues in such cases.
